# The Safety and Efficacy of Stem Cell Therapy as an Emerging Therapy for ALS: A Systematic Review of Controlled Clinical Trials

**DOI:** 10.3389/fneur.2021.783122

**Published:** 2021-12-01

**Authors:** Ammar Aljabri, Alhussain Halawani, Ghassan Bin Lajdam, Suhail Labban, Samah Alshehri, Razaz Felemban

**Affiliations:** ^1^College of Medicine, King Saud Bin Abdulaziz University for Health Sciences, Jeddah, Saudi Arabia; ^2^King Abdullah International Medical Research Center, Jeddah, Saudi Arabia; ^3^Department of Clinical Pharmacy, College of Pharmacy, King Abdulaziz University, Jeddah, Saudi Arabia

**Keywords:** amyotrophic lateral sclerosis, stem cell therapies, systematic (literature) review, motor neuron disease, regenerative medicine, controlled trials

## Abstract

Amyotrophic lateral sclerosis (ALS) is a neurodegenerative disease with a heterogeneous course that ultimately leads to death. Currently, there is no cure, and new treatments that can slow the progression of the disease are needed. Stem cell (SC) transplantation is an emerging therapy that has shown a lot of potential in recent clinical trials. This review is aimed to examine the results of various clinical trials on this topic, thus assessing the safety and efficacy of SC transplantation as a potential treatment for ALS. We identified 748 studies in our search, of which 134 full-text studies were assessed for eligibility. Six studies met the inclusion criteria and were included in this review. Although some of the included studies showed the positive effect of SC transplantation, other studies found that there was no significant difference compared to the control group. We observed more positive effects with bone marrow mesenchymal stem cells (BM-MSC) treatments than Granulocyte colony-stimulating factor (G-CSF) ones. However, other factors, such as route of administration, number of doses, and number of cells per dose, could also play a role in this discrepancy. Based on this information, we conclude that more properly conducted clinical trials are needed to appreciate the benefit of this treatment.

## Introduction

Amyotrophic lateral sclerosis (ALS) is a progressive neurodegenerative disease characterized by loss of upper and lower motor neurons, resulting in worsening weakness of voluntary muscles. ALS inevitably leads to paralysis, respiratory insufficiency, and eventually death (1). The overall prevalence and incidence of ALS are estimated at 4.42 per 100,000 and 1.59 per 100,000, respectively. The median survival time was estimated to be 4.32 years from the time of onset for the eastern European population (2).

Amyotrophic lateral sclerosis can be hereditarily classified into a familial (FALS) and a sporadic form with the latter constituting ~90% of all cases (3). The underlying pathophysiological mechanisms for ALS have been a topic of extensive research. Genetic and environmental interactions lead to the development of ALS in a potentially consecutive six-step manner (4). More than 30 genes have been identified as a major risk for the development of ALS, 4 of which account for 70% of all FALS cases, which include C9orf72, TARDBP, SOD1, and FUS (5). Moreover, brain microscopic changes in ALS depict neuronal and axonal losses. There is both reduction and loss of motor neurons, mainly in the anterior horn of the spinal cord and lower cranial motor nuclei in the brainstem (6). The key pathological hallmark of ALS is ubiquitinated bodies, which are mostly composed of TAR DNA binding protein 43 (TDP-43) proteins found in the degenerating neurons (7).

Glutamate neurotoxicity has also been implicated in the pathogenesis of ALS. Riluzole, an antiglutamatergic drug, remains the only disease-modifying therapy that successfully slows the progression of ALS and prolongs the mean patient survival by 3–6 months (8, 9). Although Riluzole slows the progression and improves survival, symptoms still appear and make the therapy more complex. In addition, non-motor symptoms, such as cognitive impairment and psychiatric illnesses, may be seen. In addition to Riluzole, a free radical scavenger known as Edaravone is used. In a study conducted in Japan, Edaravone caused a smaller decline in the revised ALS Functional Rating Scale (ALSFRS-R) in the treatment group compared to the placebo. However, the study failed to demonstrate significant efficacy (10).

Stem cell (SC) therapy is considered one of the most promising therapeutic approaches for ALS. With this therapy, various pathogenic mechanisms could be targeted to slow the progression of the disease. SC therapy could provide both trophic and immunomodulatory support and potentially allow for the regeneration of motor neurons (11). The use of SC therapy to slow the progression of ALS has been supported in many preclinical animal models (12). Many phase 1 and phase 2 trials aimed to assess feasibility and safety, with some looking at clinical benefits reflected through parameters, such as ALSFRS-R and respiratory function. However, these trials have failed to show significant results, which could be attributed to factors, such as limited sample size and lack of a control group. Furthermore, controlled studies provide a higher level of evidence. Many studies have included a control group to highlight the true benefit of SC therapy. Therefore, given the need for alternative therapy, we decided to conduct a systematic review of controlled clinical trials to determine the efficacy of SC therapy in the treatment of ALS.

## Methods

We selected controlled clinical trials, including randomized controlled trials (RCTs), quasi-randomized trials, and non-randomized clinical trials (Table 1). Patients with ALS included in the study were diagnosed using the El Escorial criteria with no age restriction. Studies were included regardless of the type of SCs used. We excluded animal studies and uncontrolled clinical trials from this study. The primary outcome for this review is the change in the rate of functional impairment measured using the ALSFRS-R. The ALSFRS-R is a questionnaire-based scale that assesses the physical ability of patients with ALS to perform activities of daily living (ADL). The questions in the scale assess four domains: gross, fine motor tasks, bulbar, and respiratory functions. Each question is scored from 0 (fully impaired) to 4 (normal) with a total of 12 questions and 48 scores (19). ALSFRS-R can be either reported solely as a score or as a slope that is interpreted as the rate of change in the ALSFRS-R score. The ALSFRS-R slope is of widespread recognition as a critical indicator of prognosis in patients with ALS (20). Secondary outcomes include respiratory function reflected by forced vital capacity (FVC), overall mortality, and adverse effects of SC therapy. For trials that used invasive sham procedures, ethical concerns regarding safety should be considered.

**Table 1:** Model for study and stem cell characteristics.

**References**	**Type**	**Patients (n)**	**Cell type**	**Flow cytometry**	**Dose: cells × 106 × times**	**Administration route**
Martinez et al. (13)	Non-randomized controlled trial	Control: 10	BM-MSC	CD133+	2.5–7.5 × 105	Subcutaneous
		Treatment: 10				
Nefussy et al. (14)	RCT	Control: 18	G-CSF	CD34+	5 mg/kg/day x 4 days	Subcutaneous
		Treatment: 39				
Amirzagar et al. (15)	RCT	Control: 20	G-CSF	CD34+/CD133+	5 μg/kg/q12h x 5 days	Subcutaneous
		Treatment: 20				
Rushkevich et al. (16)	Non-randomized controlled trial	Control: 15	1. Intact BM-MSC 2. Neural induced BM-MSC	CD29+, CD44+, CD73+, CD105+CD34–, CD45–	Intact: 0.5–1.5 × 106/kg body weight (42–102 × 106 cells) Neural induced: 5.0–9.7 × 106 cells	Intravenous and intralumbar (L3-L4)
		Treatment: 10				
Oh et al. (17)	RCT	Control: 31	BM-MSC	CD29+, CD44+, CD49+, CD73+, CD105+CD34–, CD45–	1/kg × 2 (1 month apart) in CSF	Intrathecal (L2–L4)
		Treatment: 33				
Berry et al. (18)	RCT	Control: 12	MSC-NTF	-	Combined IT (125 × 106 MSC-NTF cells in a 5-mL syringe, using a 20-G spinal needle) administration and 24 IM (48 × 106 MSC-NTF cells)	Intrathecal and intramuscular
		Treatment: 48				

**Figure 1:**
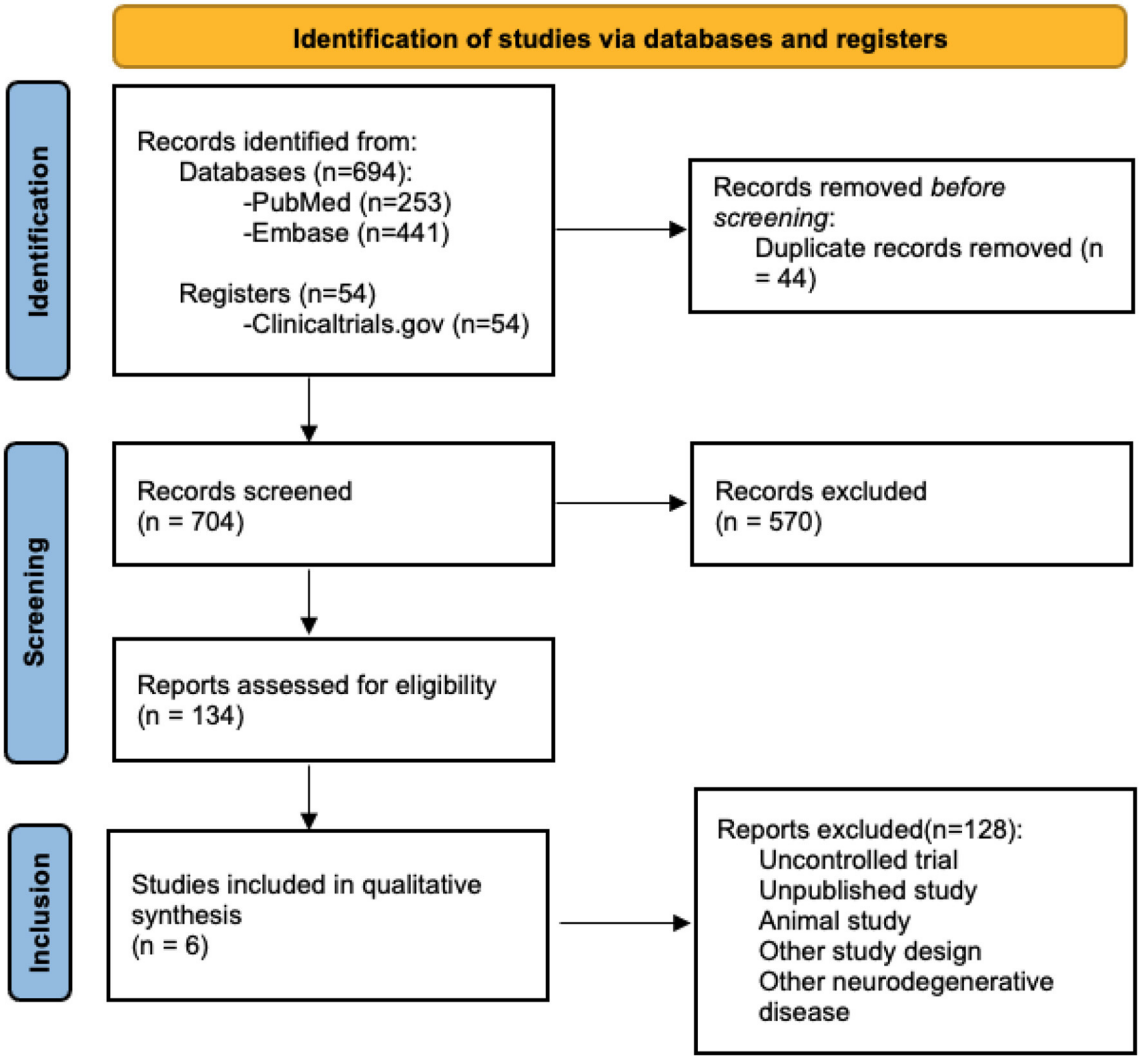
PRISMA for included studies.

We searched the following databases on April 12, 2021: Medline and Embase. In addition, we searched the US National Institutes of Health Clinical Trials Registry (www.clinicaltrials.gov). The first round of searching involved screening based on titles and abstracts. Four independent reviewers assessed each study for relevancy based on the eligibility criteria. Only full-text studies published in English and that reported numerical data for ALSFRS were included. Studies that were not excluded after the first round of searching were reassessed in a second round based on the context presented in the full article. Disagreement between the reviewers was resolved by consensus. Consultation by a third party was not needed for issue resolution. Sufficient details of the selection process were documented using a PRISMA diagram (Figure 1).

### Data Extraction

Two models for data extraction were made. The first model included information about the type of study, number of patients, type of SC, administration route, and other SC descriptors (Table 1). In the second model, numerical data of ALSFRS are provided and include mean baseline values and mean values at various follow-up points after SC therapy (Table 2). Two reviewers extracted the outcome data from the studies, and two other reviewers revised the data. Disagreements were solved by discussion and consensus.

**Table 2:** Model for ALSFRS-R mean baseline scores/slopes and mean scores/slopes after stem cell therapy at different follow-up points.

**References**	**Mean baseline ALSFRS-R score (points) or slope (points/month)**	**At 1 month**	**At 3 months**	**At 4-months**	**At 6-months**	**At 12 months**
Martinez et al. (13)	Treatment = 24.6 Control = 31.4	-	-	-	Treatment = 27.9 Control = 25.1	Treatment = 24 Control = 15.7
Nefussy et al. (14)	Treatment: 36.1 Control: 34.6	-	Treatment: 32.8 Control: 30.6	-	Treatment: 31.4 Control: 28.1	-
Rushkevich et al. (16)	Treatment = 40 Control = 41	-	-	-	-	Treatment = 34 Control = 16
Amirzagar et al. (15)	Treatment: 33.3 Control: 36.6	Treatment: 31.5 Control: 34.8	Treatment: 29.0 Control: 32.5	-	-	-
Oh et al. (17)	Treatment = 35.5 Control = 34.7	-	-	Treatment = 33.8 Control = 30	Treatment = 32.4 Control = 28.22	-
Berry et al. (18)	Treatment = 38 Control = 38.6	Treatment = +0.6 points/month Control = −0.03 points/month	-	-	-	-

## Results

### Efficacy

We examined six studies analyzing the efficacy of SC transplantation in patients with ALS. Efficacy was measured using the ALSFRS-R, which ranged from 48 (normal) to 0 (fully impaired). Included studies used the following routes of administration: subcutaneous (13–15), combined intrathecal and intramuscular approach (18), intravenous followed by intralumbar injections (16), and intrathecal approach (17). Out of the six studies, three used Granulocyte colony-stimulating factor (G-CSF) (13–15), and the remaining used bone marrow mesenchymal stem cells (BM-MSC) (16–18).

Two studies conducted trials of a 6-month follow-up period. Oh et al. observed a slower decline in ALSFRS-R with 2 BM-MSCs injections, which demonstrated clinical benefit during the period of the study (17). They also noted a significant difference in the ALSFRS-R slope between MSCs and control groups. Berry et al. observed early improvements in ALS clinical scores after a single dose with ≥1.5 points/month ALSFRS-R slope improvement in the treatment group at all times during the study (18).

The other four studies followed patients for 12 months. Rushkevich et al. demonstrated significant improvement in the mean ALSFRS-R in the treatment group (16). Similarly, Martinez et al. found that the ALSFRS-R score was significantly improved throughout the follow-up period compared to baseline (13). However, both Nefussy et al. and Amirzagara et al. did not observe a significant difference in the efficacy between treatment and placebo groups after the injections (14, 15). Mean ALSFRS-R scores for each study are provided (Table 2).

### Respiratory Function

Out of the six studies included in the review, only three reported changes in respiratory function potentially related to SC therapy (13, 14, 17). Oh et al. found that the FVC was decreased by a mean of 11.28 (10.06) in the treatment group and 10.75 (8.40) in the control group at the 4-month follow-up point (17). However, there was no significant difference between the two groups (17). Nefussy et al. reported similar results. At the 6-month follow-up point, there was a decrease in FVC by a mean of 12.25 (15.9) in the treatment group and 10.9 (20.4) in the control group (14). The difference between the groups was also not statistically significant (14). In the study of Martinez et al. a statistical comparison of FVC between the two arms could not be performed due to three cases of required ventilatory support and three cases of death in the control group (13). However, FVC remained stable at the 1-year follow-up point in the treatment group except for two patients who could not complete the functional respiratory test (13).

### Adverse Events

The safety of SC transplantation was assessed based on the occurrence of treatment-related adverse events and mortality. Oh et al. recorded the incidence of adverse events during 4- and 6-month follow-up points and found that there were no significant differences between the two groups (17). Influenza-like illness was the most commonly observed AE in the MSC group (*N* = 7). Four incidences of adverse drug reactions (ADRs) were observed throughout the study in the MSC group, which included headaches, pyrexia, and pain. The incidence of severe adverse events in the MSC group was not considered treatment related. Four deaths occurred in the study: one in the MSC group and three in the control group (17). Berry et al. observed 16 is serious adverse events (SAEs): 9 in the MSC-NTF group and 2 in the placebo group (18). All treatment-related SAEs were considered to be due to the disease progression and not the treatment. There were no deaths during the study (18).

Rushkevich et al. found no serious side effects. However, one patient experienced a fever that normalized after 2 h following intravenous injections of MSC (16). Additionally, two patients experienced post-puncture headache syndromes after transplantation (16). Seven out of 15 patients in the control group were died due to respiratory insufficiency. Martinez et al. reported a total of five deaths at the 1-year follow-up point: two occurred in the treatment group and three in the control group (13). These deaths were deemed to be because of respiratory insufficiency (13). Nefussy et al. found that the only side effects related to the drug were bone marrow and muscle pain (14). Two patients reported these side effects: one in the treatment group and one in the control group. The SAEs reported in the study included the following: dysphagia and respiratory distress, which were considered typical for disease progression and were comparable in both groups (14). No deaths were reported in the study. In the Amirzagar et al. study, only one patient experienced transient fever and chills on day 4 of treatment (15). No deaths were reported in the study.

## Discussion

Stem cell therapy is one of the most promising new approaches in the treatment of ALS. It is proposed to target various mechanisms to slow disease progression and improve the overall quality of life. Here, we conducted a systematic review to determine the efficacy and safety of SC therapy in improving outcomes for patients with ALS. First, the change in ALSFRS-R was used to determine the efficacy of SC therapy, which is considered to decline linearly for the majority of diseases (21). For all included studies, SC therapy had a positive effect in slowing the progression of the disease, as evidenced by the difference in the ALSFRS-R score between the treatment and control groups. However, for two of the studies, the effect was not statistically significant.

All three studies that administered BM-MSC observed a significant decrease in the progression of disease burden with an overall slower decline in the ALSFRS-R score. Two studies that used G-CSF did not observe a significant benefit. Although Martinez et al. observed a significant effect of G-CSF administration, the study included only 10 patients in the treatment group and a drop out of 50%. Moreover, positive outcomes were seen in studies that involved the intrathecal administration of SCs. Both studies that did not observe significant results used subcutaneous injections. Lastly, included studies used various follow-up periods. All studies that followed patients for 6 months observed significant differences in ALSFRS-R between the treatment and control arms. One of which noted that changes in the ALSFRS-R slopes between treatment groups were similar after the first 2 months of the study. For the other four studies where the follow-up period was 12 months, three of which did not provide reliable evidence of the clinical benefit of SC therapy. Therefore, SC therapy demonstrated a transient benefit in slowing the progression of the illness, particularly, intrathecal-administered MSCs. This is supported by a recent meta-analysis that included 11 studies that showed that SC therapy demonstrated a short-term benefit (22). It is imperative to consider the limitation of included studies in determining the efficacy of SC therapy. First, the follow-up period is short, making it difficult to determine the long-term effects of such therapy. Second, the long-term benefits of the intervention must be interpreted with caution due to the loss of patients during follow-up and thus excluded from the final analysis.

The original notion behind the use of SCs to treat ALS was to employ SCs or neural progenitor cells to generate motor neurons lost with disease progression. However, this concept was difficult to implement (23, 24). For this theory to work, injected SCs need to generate motor neurons to be integrated with the pre-existing neural circuit and project axons and synapse with other neurons and muscles. The beneficial role of SCs in ALS could be explained by the “neighborhood” theory where SCs target the toxicity of neighboring cells. Moreover, transplanted cells take on a supportive role by secreting neurotrophic factors and improving the motor microenvironment of neurons, thereby delaying the disease progression.

There are many sources of SCs used in ALS trials. This review only included G-CSF-induced peripheral blood SCs and BM-MSCs. Firstly, G-CSF works by mobilizing BM CD34+ hematopoietic SCs and generating PBSCs in the peripheral bloodstream. In preclinical animal trials, G-CSF was found to stimulate PBSCs production and penetrate into the central nervous system where they generated neural cells (25). G-CSF is administered subcutaneously. It is non-invasive and does not require immunosuppression, limiting the risk of infections. However, the downside of such an intervention is the lack of evidence regarding central nervous system (CNS) penetration. Secondly, BM-MSCs are another source of SCs used in many trials. These cells are multipotent and retain the ability to differentiate into many cell types. The use of BM-MSCs has shown promising results in preliminary studies with their ability to generate a neuroprotective environment by releasing different factors (26, 27). As demonstrated by various studies, BM-MSCs can differentiate into variable cells, including neurons and glial cells. Similar to G-CSF, BM-MSCs do not require immunosuppression because they are autologous (28, 29).

The introduction of SCs into the CNS comes with many challenges. Delivering therapeutic doses to target upper motor neurons, lower motor neurons, or spinal cord requires multiple injections, which increases the risk of AEs. Particularly with interventions that target the brain, a careful risk-to-benefit analysis must be conducted. Moreover, choosing the appropriate route of injection is equally important. Many trials have supported intrathecal injections of SCs as safe and efficacious (17, 30–32). It allows for better distribution and potentially increases the delivery of cells into the subarachnoid space and access to the brain parenchyma. The intrathecal approach also allows repeated injections of SCs to be considered a minimally invasive procedure with a good side effect profile. Microglial cells have been implicated in the progression of ALS by accelerating neuronal death. Intrathecal MSCs have been proposed to induce an anti-inflammatory state, particularly by switching the phenotype of microglial cells from the proinflammatory M1 to the anti-inflammatory M2 (33). On the other hand, despite being considered safe, the intravenous delivery of MSCs has been found to have modest clinical outcomes compared to other routes (34). Therefore, all of these variables must be considered in developing an evidence-based protocol before the initiation of such treatment in patients with ALS.

We also examined the effect of SC therapy on FVC in this review. FVC was found to progress in a trajectory manner with three main trajectories: stable low, rapid progressor, and slow progressor (35). Due to these differences, it is a less reliable measure of treatment benefit. Two of the included studies analyzed differences in FVC between the two arms. Although not statistically significant, both studies showed a higher decline in FVC for the treatment groups. A possible explanation for this phenomenon could be the disturbance of the respiratory function by SC injections. This is similar to the acceleration of disease progression observed with surgery in ALS patients (36). However, SC therapy is a minor procedure, which could indicate some potential off-target effects of injected SCs as another reason for this phenomenon.

Early clinical trials have made great progress in delineating the safety of SC therapy in the treatment of ALS. Current studies on this topic seem to lack a unified approach, whether it will be the study design or the lack of agreed-upon inclusion criteria. The question of critical importance includes determining how effective SCs are compared to other forms of therapy. It is also equally important to determine the answer to the following questions: what type of SCs produces better outcomes?; what is the appropriate method and dose? and which approach is the safest? Moreover, outlining the therapeutic window is also crucial, and this includes knowing who will benefit the most from such therapy and if it is only an option in the early period of the disease. Future studies should subanalyze their patients into various disease variants according to their presentation due to the heterogeneity of the disease. The current data of SC therapy hold great promise, but more properly designed clinical trials are needed to truly verify their benefit.

## Data Availability Statement

The original contributions presented in the study are included in the article/supplementary material, further inquiries can be directed to the corresponding author/s.

## Author Contributions

AA contributed to conducting the search strategy and writing the manuscript. RF supervised the research project. SA contributed to writing methodology and editing the manuscript. AH, GB, and SL participated in writing the manuscript and the data extraction process. All authors contributed to the article and approved the submitted version.

## Conflict of Interest

The authors declare that the research was conducted in the absence of any commercial or financial relationships that could be construed as a potential conflict of interest.

## Publisher's Note

All claims expressed in this article are solely those of the authors and do not necessarily represent those of their affiliated organizations, or those of the publisher, the editors and the reviewers. Any product that may be evaluated in this article, or claim that may be made by its manufacturer, is not guaranteed or endorsed by the publisher.
